# Efficacy of Ultrasound‐Guided Tibial Nerve Block Combined With Shock Wave in the Treatment of Plantar Fasciitis: A Retrospective Cohort Study

**DOI:** 10.1155/prm/9045017

**Published:** 2026-09-26

**Authors:** Guanghui Zhang, Xin Yao, Zhiji Chen, Kai Luo, Zelin Weng, Shaofang Cai, Guanjin Luo, Meisu Lin, Lifang Lu, Jing Yang

**Affiliations:** ^1^ Department of Pain Medicine, The Second Affiliated Hospital of Xiamen Medical College, Xiamen 361021, Fujian, China; ^2^ Department of Science and Education, The Second Affiliated Hospital of Xiamen Medical College, Xiamen 361021, Fujian, China; ^3^ Department of Rehabilitation Medicine, The Second Affiliated Hospital of Xiamen Medical College, Xiamen 361021, Fujian, China; ^4^ Department of Emergency Medicine, The Second Affiliated Hospital of Xiamen Medical College, Xiamen 361021, Fujian, China

**Keywords:** extracorporeal shockwave therapy, injections, nerve block, plantar fasciitis, retrospective studies, tibial nerve

## Abstract

**Objective:**

To evaluate the effectiveness of ultrasound‐guided tibial nerve block (TNB) with extracorporeal shock wave therapy (ESWT) versus ESWT alone or ESWT with fascial injection (FI) in plantar fasciitis (PF) of various courses.

**Methods:**

This retrospective cohort study enrolled 209 PF patients (101 acute, 108 chronic), categorized into three groups: ESWT alone (Group A), ESWT‐FI (Group B), and ESWT‐TNB (Group C). Pain intensity and foot function were assessed via visual analogue scale (VAS) and American Orthopaedic Foot‐Ankle Society (AOFAS) scores at baseline and 1‐, 3‐, and 6‐month follow‐ups. Subgroup analysis was performed stratified by calcaneal spur size. Multiple linear regression was applied to identify independent predictors of pain relief, and subgroup analysis stratified by calcaneal spur size was further performed to characterize treatment response across spur‐length strata.

**Results:**

For acute PF, Groups B/C yielded lower 1‐month VAS vs A (mean difference = 1.094, 95% CI 0.759–1.429, *p* < 0.001; mean difference = 1.414, 95% CI 1.088–1.739, *p* < 0.001), with no intergroup differences at 6 months (all *p* > 0.05). In chronic PF, Groups B/C maintained superior VAS/AOFAS improvements at all timepoints; and Group C showed the largest 1‐month VAS reduction vs A (mean difference = 2.998, 95% CI 2.662–3.333, *p* < 0.001). Regression identified calcaneal spur length as an independent negative predictor of pain relief (standardized *β* = −0.166, 95%CI −0.090−0.031, *p* < 0.001). Subgroup analysis revealed Group C optimal for spurs < 2.5 mm, comparable efficacy of B/C for 2.5–5 mm spurs, and superior ΔVAS in Group B for spurs > 5 mm. Only two transient heel numbness and five transient post‐ESWT pain flares occurred, without severe adverse events.

**Conclusion:**

For chronic PF, ESWT combined with interventional therapy (especially TNB) provides greater clinical benefit. Heel spur length can serve as a predictor to guide individualized combination therapy (fascial injection or nerve block).

## 1. Introduction

Plantar fasciitis (PF) is the most common cause of heel pain; its pathological nature is plantar fascia attachment points having repeatedly more microdamage and degenerative changes, rather than to simply aseptic inflammation Although most patients respond well to conservative treatment [1], about 10% of the cases will be turned into intractable pain, illnesses out, seriously affecting the quality of life [2].

Extracorporeal shock wave therapy (ESWT) has been used extensively in clinical practice as an effective means to facilitate tissue repair [3–6]. However, a single mode in intractable pain curative effect is studied [7, 8]. In recent years, the accuracy of ultrasound‐guided interventional techniques, such as plantar fascia around the injection (FI) and tibial nerve block (TNB), has been studied because of its targeted delivery drugs or block pain signaling, showing good application prospects [9–12]. In theory, TNB by blocking plantar sensory nerve, main can build better analgesia environment and create favorable conditions for tissue repair.

Currently, there is a lack of direct comparative studies on the combination of ESWT and different intervention modes (FI and TNB), as well as a lack of predictive analysis of efficacy for specific imaging characteristics (such as heel spur) [13, 14]. Therefore, this study aims to study the retrospective cohort analysis, compare the clinical curative effect of three kinds of treatments, and heel spur length value in guiding individualized treatment.

## 2. Material and Methods

### 2.1. Study Subjects and Ethical Statement

The retrospective study was approved by the Medical Ethics Committee of The Second Affiliated Hospital of Xiamen Medical College (Approval No.: 2025242). Since the study was retrospective, the informed consent requirement was waived. We set a particular period of patient recruitment between January 2023 and January 2025. All consecutive cases that satisfied the inclusion criteria within this time were recruited.

Inclusion criteria: (1) Patients had diagnostic criteria of PF, which comprised the typical first step pain in the morning and tenderness at the medial calcaneal node trigger point [1, 15]; (2) age of 18–70 years; (3) course record is complete and completed 6 months of follow‐up; and (4) patients who had previously received conservative treatments (such as physiotherapy, orthotics, or corticosteroid injections) or were on regular analgesic medication for PF were excluded to ensure treatment naivety.

We also obtained and corrected the height, weight, BMI, history of diabetes, smoking status, and time spent standing/walking per day (divided into 4 h less than 4 h, 4–8 h, more than 8 h) to avoid confounding in multivariable analysis.

Exclusion criteria: (1) previous history of foot surgery, fracture, tumor, or infectious lesion; (2) pregnancy or lactation women; (3) with severe disease of heart head blood vessel, blood coagulation dysfunction, or systemic autoimmune disease; and (4) there are local injections or shock wave therapy contraindicated (such as skin burst at the injection site, severe diabetes peripheral neuropathy).

### 2.2. Sample Grouping and Stratification

The clinical data of 209 patients were finally included. According to the course of the disease, it is bounded (3 months) the patients are divided into acute course groups (< 3 months, *n* = 101) with the chronic course group (≥ 3 months, *n* = 108). Patients were further grouped into three groups according to the actual treatment regimens they had received:

Group A (ESWT‐only group): Received ESWT alone.

Group B (FI + ESWT group): Received a combination of ultrasound‐guided perifascial injection and ESWT.

Group C (TNB + ESWT group): Received a combination of ultrasound‐guided TNB and ESWT.

**FIGURE 1:**
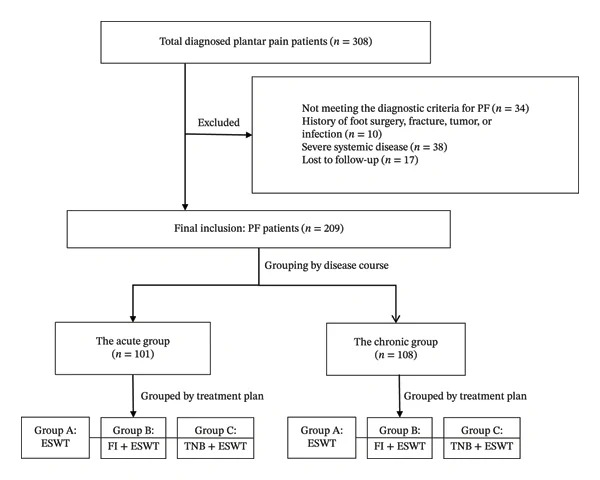
Flowchart of patient selection, enrollment, and treatment allocation.

There was no statistically significant difference in age, gender, BMI, and baseline pain score between the three groups (all *p* > 0.05) and good comparability among groups (Figure 1).

### 2.3. Treatment Methods

Shared decision‐making was used to choose the treatment based on the preference of invasiveness, economic considerations, and previous adherence between the attending physician and the patient. All procedures were performed by the same pain specialist with > 5 years of experience in musculoskeletal ultrasound.

#### 2.3.1. Ultrasound‐Guided Interventions

Ultrasound guidance was performed using a Mindray Resona R9 system with a high‐frequency linear probe (L20‐5). Patients were placed prone with the foot relaxed.

Group A received no injection.

In the case of Group B (FI + ESWT), the needle was inserted in‐plane to the subfascial space at the medial calcaneal tubercle. Negative aspiration was done, and then the same 3‐mL injectate was administered.

In the case of Group C (TNB + ESWT), tibial nerve was found near the posterior tibial vessels at the tarsal tunnel. A 25‐gauge x 38‐mm spinal needle was inserted using an in‐plane technique, and 3 mL of injectate was injected following negative aspiration. The injectate was made up of 0.4% lidocaine, 0.5 mL of betamethasone (4 mg), 0.5 mL of mecobalamin (0.5 mg), and 0.5 mL of normal saline.

#### 2.3.2. ESWT

All patients received radial ESWT using a Swiss DolorClast Smart device (EMS, Nyon, Switzerland). The energy flux density was determined as 0.15–0.25 mJ/mm^2^ (medium‐low range according to the ISMST classification). It provided 2000 pulses at the rate of 5–8 Hz and 2.5–4.0 bar in each session once weekly over a period of 5 weeks.

#### 2.3.3. Rehabilitation Protocol

To minimize performance bias, all patients were instructed to perform a standardized plantar fascia stretching regimen (3 sessions daily, 10 min per session) during treatment and follow‐up. No additional formal physical therapy was permitted. Treatment parameters: the frequency was set at 5–8 Hz, the pressure was adjusted to 2.5–4.0 bar (250∼400 kPa), and the energy flow density was controlled within the safe and effective range of 0.15–0.25 mJ/mm^2^. Each session consisted of 2000 pulses. The pressure was adjusted during treatment based on patient tolerance. Treatments were administered once per week for five consecutive weeks [16].

### 2.4. Outcome Measures

The two most important measures of the outcome were pain score and function score that were measured prior to treatment (baseline), 1 month (short‐term) post‐treatment, 3 months (medium‐term) post‐treatment, and 6 months (long‐term) post‐treatment.

#### 2.4.1. Pain Intensity

By the method of visual analogue scale (VAS, 0–10), patients themselves evaluate the level of rest and load pain when walking.

#### 2.4.2. Functional Status

American Orthopaedic Foot and Ankle Society (AOFAS) ankle‐hindfoot function scale (100 points) for pain, function, and radiographic expression.

#### 2.4.3. Radiographic Subgroup Analysis (Chronic Group Only)

Based on pretreatment lateral foot radiographs, the maximum vertical length of the calcaneal spur was measured. The chronic patients were then stratified into three subgroups, i.e., spur < 2.5 mm, 2.5–5.0 mm, and above 5.0 mm. This analysis used a change in VAS score at baseline (ΔVAS) as the efficacy index.

#### 2.4.4. Clinical Efficacy

Secondary outcomes included the Modified MacNab Criteria and patient subjective satisfaction (very satisfied, satisfied, dissatisfied) at 3 and 6 months.

### 2.5. Statistical Analysis

The sample size was calculated a priori with G power 3.1. Under the assumption that = 0.05, power = 0.80, an anticipated difference in VAS of 1.0, and a standard deviation of 1.5, at least 27 patients per group were needed; 209 patients were recruited to allow any dropout. The statistical analysis was done through SPSS 27.0. No missing data were found across all analytical variables; thus, statistical analyses were performed on the complete dataset without missing‐value imputation. The Shapiro–Wilk test was used to test normality of continuous variables. Data normally distributed were presented as mean standard deviation (Mean ± SD); non‐normally distributed data were presented as median [interquartile range] (Median [IQR]). Categorical variables were reported as the number of cases (percentage) [*n* (%)]. All statistical tests were two‐sided, and *p* < 0.05 was taken to be statistically significant. Normally distributed continuous variables were subjected to one‐way analysis of variance (ANOVA) with the post hoc pairwise comparisons using the Bonferroni method. The Kruskal–Wallis H rank‐sum test was used in the analysis of non‐normal continuous variables. In the case of categorical variables, Pearson 2 test or Fisher exact test was implemented where applicable. The multiple linear regression was built with the Enter method, where Delta VAS was the dependent variable and age, sex, disease duration, heelspur length, diabetes mellitus, smoking history, daily standing time, baseline VAS, baseline AOFAS, and stratified BMI were independent variables. The model fit was tested as a whole by means of the model *F*‐test, and adjusted *R* ^2^ was applied to indicate the degree of outcome variance explained by the model. Regression coefficient B along with its 95% confidence interval was used to measure the independent effect of each variable. The two‐way repeated measures ANOVA was done in the case of a repeatedly measured VAS score and joint functioning score. In the case when there was a statistically significant interaction between group by time, then simple‐effect analysis was also carried out to examine (1) the difference between groups in the three groups at any point in time and (2) differences within groups across the time points. When it came to the nonsignificant interaction, the main effects of group and time were only reported. The Bonferroni method corrected all pairwise comparisons. The Pearson chi‐squared test was applied in remission rate and patient‐satisfaction analysis; the Fisher exact test was used when theoretical frequency of any cell was less than 5. The Bonferroni correction was used in pairwise comparison between rates of multiple groups.

## 3. Results

### 3.1. Comparison of Clinical Efficacy Between Groups

Baseline characteristics did not differ significantly among the treatment groups (*p* > 0.05, Table 1).

**TABLE 1:** Characteristics of acute and chronic plantar fasciitis patients.

Characteristic	Acute (< 3 months, *n* = 101)			*p* value	Chronic (≥ 3 months, *n* = 108)			*p* value
	Group A (*n* = 40)	Group B (*n* = 29)	Group C (*n* = 32)		Group A (*n* = 36)	Group B (*n* = 37)	Group C (*n* = 35)	
Age (years), Mean ± SD	47.18 ± 11.57	50.17 ± 11.92	50.78 ± 13.22	0.406	36.00 ± 8.37	48.46 ± 10.34	48.19 ± 9.65	0.187
Male, (*n* %)	19 (47.5)	18 (62.1)	17 (53.1)	0.488	12 (33.3)	18 (48.6)	16 (45.7)	0.376
Height (cm)	163.65 ± 6.74	164.72 ± 8.24	164.75 ± 8.68		163.64 ± 8.15	164.62 ± 7.28	163.77 ± 8.52	
Weight (kg)	65.10 ± 9.98	66.24 ± 9.80	67.06 ± 9.28		65.17 ± 9.94	65.68 ± 9.58	64.51 ± 11.35	
BMI				0.842				0.873
Low risk for obesity (below 18.5)	3	0	0		0	1	1	
Risk for nutritional deficiency (18.5–22.9)	9	9	8		14	11	12	
Moderate risk for obesity (23–27.4)	23	17	21		20	22	17	
High risk for obesity (27.5 and above)	5	3	3		2	3	5	
Upright time				0.635				0.271
< 4 hour	9	8	9		9	4	11	
4–8 hour	24	13	14		14	20	14	
> 8 hour	7	8	9		13	13	10	
Diabetic status				0.986				0.884
NO	7	5	6		2	3	2	
YES	33	24	26		34	34	33	
Smoking status				0.889				0.671
NO	31	21	24		31	29	28	
YES	9	8	8		5	8	7	
Symptom Duration (months), Median (IQR)	1.00 (2)	1.00 (1)	2.00 (1)	0.054	6.00 (17)	6.00 (8)	12.00 (9)	0.086
Heel Spur Length (mm), Median (IQR)	0.95 (5.2)	3.10 (4.8)	1.95 (4.8)	0.406	3.30 (5.9)	2.80 (4.6)	2.90 (6.0)	0.381
Heel Spur Present, *n* (%)	24 (60.0)	22 (75.9)	20 (62.5)	0.361	26 (72.2)	23 (62.2)	27 (77.1)	0.363
Baseline VAS Score, Mean ± SD	5.51 ± 0.69	5.43 ± 0.60	5.70 ± 0.92	0.333	5.65 ± 0.79	5.67 ± 0.76	5.57 ± 0.78	0.873
Baseline AOFAS Score, Mean ± SD	55.50 ± 8.89	53.62 ± 7.10	55.22 ± 8.27	0.545	35.89 ± 8.05	37.73 ± 8.37	35.06 ± 8.14	0.368

#### 3.1.1. Changes in VAS Scores

##### 3.1.1.1. Acute Patients (Disease Duration < 3 Months)

In the case of patients with the disease period less than 3 months, the analysis of VAS in a repeated‐measures ANOVA was not found to be sphericity (*W* = 0.177, *p* < 0.001), and therefore, the Greenhouse–Geisser correction was used to correct degrees of freedom. The results showed that time had significant main effects (*F* (1.490, 146.015) = 6583.926, *p* < 0.001, partial *η* ^2^ = 0.985) and group (*F* (2, 98) = 13.560, *p* < 0.001, partial *η* ^2^ = 0.217) as well as a statistically significant interaction between time and group (*F* (2.980, 146.015) = 61.599, *p* < 0.001, partial *η* ^2^ = 0.557). Due to the importance of the interaction effect, it was further broken down by performing simple effects tests.

Between‐group comparisons: Simple effects analysis revealed statistically significant intergroup differences in VAS scores at 1‐month (*F* (2, 98) = 92.938, *p* < 0.001) and 3‐month (*F* (2, 98) = 34.144, *p* < 0.001) post‐treatment, whereas no statistically significant intergroup differences were observed at baseline or at 6‐month post‐treatment (all *p* > 0.05).

Post hoc pairwise comparisons with the Bonferroni correction yielded the following results:

At 1‐month post‐treatment: VAS scores in Group A were significantly higher than those in Group B (95% CI: 0.759–1.429, *p* < 0.001) and Group C (95% CI: 1.088–1.739, *p* < 0.001); no statistically significant difference was detected between Group B and Group C (*p* > 0.05).

At 3‐month post‐treatment: VAS scores in Group A remained significantly higher than those in Group B (95% CI: 0.232–0.745, *p* < 0.001) and Group C (95% CI: 0.588–1.008, *p* < 0.001). A statistically significant difference was also observed between Group B and Group C, with Group B reporting higher VAS scores (95% CI: 0.080–0.620, *p* = 0.006).

At 6‐month post‐treatment: No statistically significant pairwise differences in VAS scores were observed across the three groups (all *p* > 0.05).

Within‐group comparisons: For all three groups, VAS scores at each post‐treatment follow‐up timepoint (1, 3, and 6 months) were significantly lower relative to their respective baseline values (all *p* < 0.05) (Table 2).

**TABLE 2:** Outcomes at different time points, stratified by symptom duration.

**Symptom duration/outcome**	**Time point**			
**Acute (< 3 months)**		**Group A (*n* = 40)**	**Group B (*n* = 29)**	**Group C (*n* = 32)**

VAS Score, Mean ± SD	Baseline	5.51 ± 0.69	5.43 ± 0.60	5.70 ± 0.92
	1 month	2.77 ± 0.72^∗^	1.68 ± 0.37^∗#^	1.36 ± 0.48^∗#$^
	3 months	1.19 ± 0.53^∗^	0.70 ± 0.46^∗#^	0.35 ± 0.23^∗#$^
	6 months	0.44 ± 0.23^∗^	0.40 ± 0.21^∗^	0.35 ± 0.15^∗^
		*F* time (1.490, 146.015) = 6583.926, *p* time **< 0.001**		
		*F* group (2, 98) = 13.560, *p* group **< 0.001**		
		*F* time^∗^ group (2.980, 146.015) = 61.599, *p* time^∗^ group **< 0.001**		

AOFAS Score, Mean ± SD	Baseline	55.50 ± 8.89	53.62 ± 7.10	55.22 ± 8.27
	1 month	65.60 ± 11.86^∗^	75.00 ± 7.97^∗#^	86.2 ± 6.36^∗#$^
	3 months	89.68 ± 4.75^∗^	90.31 ± 4.99^∗^	92.16 ± 3.65^∗^
	6 months	93.98 ± 1.82^∗^	94.00 ± 2.32^∗^	95.00 ± 1.93^∗^
		*F* time (1.176, 115.201) = 2053.332, *p* time **< 0.001**		
		*F* group (2, 98) = 9.482, *p* group **< 0.001**		
		F time^∗^ group (2.351, 115.201) = 61.599, *p* time^∗^ group **< 0.001**		

**Chronic (≥ 3 months)**		**Group A (*n* = 36)**	**Group B (*n* = 37)**	**Group C (*n* = 35)**

VAS Score, Mean ± SD	Baseline	5.65 ± 0.79	5.67 ± 0.76	5.57 ± 0.78
	1 month	4.99 ± 0.76	2.70 ± 0.44^∗#^	1.99 ± 0.50^∗#$^
	3 months	3.35 ± 0.54^∗^	2.50 ± 0.51^∗#^	2.22 ± 0.79^∗#^
	6 months	2.40 ± 0.62^∗^	1.08 ± 0.56^∗#^	1.09 ± 0.56^∗#^
		*F* time (1.622, 170.319) = 3450.729, *p* time **< 0.001**		
		*F* group (2, 105) = 55.196, *p* group **< 0.001**		
		*F* time^∗^ group (3244, 146.015) = 159.085, *p* time^∗^ group **< 0.001**		

AOFAS Score, Mean ± SD	(ΔVAS) at 3M	2.30 ± 0.63^∗^	3.17 ± 0.35^∗#^	3.35 ± 0.79^∗#^
	Baseline	35.89 ± 8.05	37.73 ± 8.37	35.06 ± 8.14
	1 month	39.78 ± 9.35	55.24 ± 11.22^∗#^	67.63 ± 9.17^∗#$^
	3 months	56.64 ± 10.46^∗^	79.70 ± 9.28^∗#^	88.00 ± 6.46^∗#$^
	6 months	87.00 ± 2.45^∗^	93.32 ± 4.04^∗#^	94.80 ± 3.19^∗#^
		*F* time (1.188, 124.718) = 4766.876, *p* time **< 0.001**		
		*F* (2, 105) = 47.485, *p* group **< 0.001**		
		*F* time^∗^ group (2.376, 124.718) = 169.343, *p* time^∗^ group **< 0.001**		

*Note:* ΔVAS was calculated as (Baseline VAS ‐3‐month VAS). Data are presented as Mean ± SD. Bold values indicate statistically significant differences with (*p* < 0.05).

^∗^Significant as *p* value < 0.05.

^#^Differences exist between the group and Group A.

^$^Differences exist between the group and Group B.

##### 3.1.1.2. Chronic Patients (Disease Duration ≥ 3 Months)

For patients with symptom duration > 3 months, repeated‐measures ANOVA of VAS scores demonstrated that Mauchly’ s test of sphericity was violated (*W* = 0.128, *p* < 0.001); thus, the Greenhouse–Geisser correction was applied to adjust the degrees of freedom. The analysis revealed statistically significant main effects of time (*F* (1.622, 170.319) = 3450.729, *p* < 0.001, partial *η* ^2^ = 0.970) and group (*F* (2, 105) = 55.196, *p* < 0.001, partial *η* ^2^ = 0.513), alongside a significant time × group interaction (*F* (3.244, 146.015) = 159.085, *p* < 0.001, partial *η* ^2^ = 0.752). Given the significance of the interaction, subsequent simple effects analyses were performed to decompose the effect.

Between‐group comparisons: Simple effects analysis revealed statistically significant intergroup differences in VAS scores at 1‐month (*F* (2, 105) = 259.484, *p* < 0.001), 3‐month (*F* (2, 105) = 31.819, *p* < 0.001), and 6‐month (*F* (2, 105) = 62.219, *p* < 0.001) post‐treatment.

Post hoc pairwise comparisons with the Bonferroni correction yielded the following results:

At 1‐month post‐treatment: VAS scores in Group A were significantly higher than those in Group B (95% CI: 1.955–2.617, *p* < 0.001) and Group C (95% CI: 2.662–3.333, *p* < 0.001); VAS scores in Group B were also significantly higher than those in Group C (95% CI: 0.378–1.045, *p* < 0.001).

At 3‐month post‐treatment: VAS scores in Group A remained significantly higher than those in Group B (95% CI: 0.498–1.207, *p* < 0.001) and Group C (95% CI: 0.767–1.487, *p* < 0.001); no statistically significant difference was detected between Group B and Group C (*p* < 0.05).

At 6‐month post‐treatment: VAS scores in Group A were significantly higher than those in Group B (95% CI: 0.995–1.654, *p* < 0.001) and Group C (95% CI: 0.975–1.642, *p* < 0.001); no statistically significant difference was observed between Group B and Group C (*p* < 0.05).

Within‐group comparisons: For all three groups, VAS scores at each post‐treatment follow‐up timepoint (1, 3, and 6 months) were significantly lower relative to their respective baseline values (all *p* < 0.05).

#### 3.1.2. Changes in AOFAS Scores

##### 3.1.2.1. Acute Patients (Disease Duration < 3 Months)

Repeated‐measures ANOVA of AOFAS ankle‐hindfoot scores indicated that Mauchly’ s test of sphericity was violated (*W* = 0.025, *p* < 0.001); thus, the Greenhouse–Geisser correction was applied to adjust degrees of freedom. The analysis revealed statistically significant main effects of time (*F* (1.176, 115.201) = 2053.332, *p* < 0.001, partial *η* ^2^ = 0.954) and group (*F* (2, 98) = 9.482, *p* < 0.001, partial *η* ^2^ = 0.162), alongside a significant time × group interaction (*F* (2.351, 115.201) = 61.599, *p* < 0.001, partial *η* ^2^ = 0.537). Given the significance of the interaction, subsequent simple effects analyses were performed.

Between‐group comparisons: Simple effects analysis revealed statistically significant intergroup differences in AOFAS scores at 1‐month post‐treatment (*F* (2, 98) = 43.607, *p* < 0.001), whereas no statistically significant intergroup differences were observed at baseline or at 3‐ and 6‐month post‐treatment (all *p* < 0.05).

Post hoc pairwise comparisons with Bonferroni correction yielded the following results:

At 1‐month post‐treatment: Group A had significantly lower AOFAS scores than Group B (95% CI: −14.949 ∼ −3.861, *p* < 0.001) and Group C (95% CI: −26.036 ∼ −15.264, *p* < 0.001); Group B also had significantly lower AOFAS scores than Group C (95% CI: −17.073 ∼ −5.427, *p* < 0.001).

At 3‐ and 6‐month post‐treatment: No statistically significant pairwise differences in AOFAS scores were observed across the three groups (all *p* < 0.05).

Within‐group comparisons: For all three groups, AOFAS scores at each post‐treatment follow‐up timepoint (1, 3, and 6 months) were significantly improved relative to their respective baseline values (all *p* < 0.05).

##### 3.1.2.2. Chronic Patients (Disease Duration ≥ 3 Months)

Mauchly’ s test of sphericity was violated for AOFAS scores in patients with symptom duration > 3 months (*W* = 0.039, *p* < 0.001), prompting the Greenhouse–Geisser correction. Statistically significant main effects of time (*F* (1.188, 124.718) = 4766.876, *p* < 0.001, partial *η* ^2^ = 0.978) and group (*F* (2, 105) = 47.485, *p* < 0.001, partial *η* ^2^ = 0.475) were observed, as well as a significant time × group interaction (*F* (2.376, 124.718) = 169.343, *p* < 0.001, partial *η* ^2^ = 0.763). Subsequent simple effects analyses were conducted to further characterize the interaction.

Between‐group comparisons: Simple effects analysis revealed statistically significant intergroup differences in AOFAS scores at 1‐month (*F* (2, 105) = 69.530, *p* < 0.001), 3‐month (*F* (2, 105) = 118.669, *p* < 0.001), and 6‐month (*F* (2, 105) = 56.425, *p* < 0.001) post‐treatment. Note: The third timepoint was duplicated as “3‐month post‐treatment” in the original text; it has been corrected to “6‐month post‐treatment” here to align with the subsequent comparative results, with no alteration to the original statistical values.

Post hoc pairwise comparisons with the Bonferroni correction yielded the following results:

At 1‐month post‐treatment: AOFAS scores in Group A were significantly lower than those in Group B (95% CI: −21.147–−9.784, *p* < 0.001) and Group C (95% CI: −33.612–−22.089, *p* < 0.001). Scores in Group B were also significantly lower than those in Group C (95% CI: −18.108–−6.663, *p* < 0.001).

At 3‐month post‐treatment: AOFAS scores in Group A remained significantly lower than those in Group B (95% CI: −28.141–−17.986, *p* < 0.001) and Group C (95% CI: −36.509–−26.213, *p* < 0.001). Scores in Group B were again significantly lower than those in Group C (95% CI: −13.411–−3.183, *p* < 0.001).

At 6‐month post‐treatment: AOFAS scores in Group A were significantly lower than those in Group B (95% CI: −8.204–−4.445, *p* < 0.001) and Group C (95% CI: −9.706–−5.894, *p* < 0.001). No statistically significant difference was detected between Group B and Group C (all *p* < 0.05).

Within‐group comparisons: Within‐group comparisons demonstrated statistically significant reductions in VAS scores at all post‐treatment follow‐up timepoints (1, 3, and 6 months) relative to respective baselines across Groups A, B, and C (all *p* < 0.05).

The ANOVA was conducted to compare the difference in scores between VAS and ANOVA in patients with symptom duration more than 3 months. The Levene test of homogeneity of variances did not hold. There was a significant result in the robust test of equality of means, which is *p* < 0.001 as it proves that there is heterogeneity among the groups in terms of mean values of the VAS. In line with this, the Games–Howell post hoc test, which can be used to conduct pairwise comparisons when the assumption of homogeneity of variances is violated, was used to make further pairwise comparisons. It has been found that statistically significant differences exist between Groups A and B and between Groups A and C but not between Groups B and C (all *p* < 0.05).

In the case of confounding due to nonrandomization, a multivariable linear regression was done on patients with chronic PF. The outcome measure in this research was the difference between the VAS score (ΔVAS = baseline ∼ 3‐month VAS). There were also independent variables that comprised age, sex, BMI, duration of symptoms, length of heel spur, history of diabetes, smoking, time spent standing, and the treatment group. Multicollinearity was not present as the variance inflation factors were less than 2.5.

Notably, the heel spur length was a significant independent predictor of ΔVAS (*B* = −0.071, 95% CI: −0.111–−0.029, *p* < 0.001; standardized *β* = −0.34), indicating that longer spurs were associated with smaller pain reduction. Treatment group remained significant after adjustment (*p* < 0.001) (Table 2).

#### 3.1.3. MCID Achievement Rates at 1‐Month Post‐Treatment

The proportions of patients achieving the VAS MCID (improvement ≥ 1.5 points) [17] and AOFAS MCID (improvement ≥ 9.5 points) [18] at 1‐month post‐treatment were compared among the three groups.

Among patients with symptom duration < 3 months, no statistically significant intergroup difference was observed in the VAS MCID achievement rate. In contrast, a statistically significant intergroup difference was detected in the AOFAS MCID achievement rate.

Pairwise comparisons revealed that the AOFAS MCID achievement rate in Group A was significantly lower than that in Groups B and C, while no statistically significant difference was observed between Group B and Group C.

Among patients with symptom duration ≥ 3 months, statistically significant overall intergroup differences were observed for both the VAS MCID and AOFAS MCID achievement rates. Post hoc pairwise comparisons demonstrated that the proportions of patients achieving both VAS and AOFAS MCID were significantly higher in Groups B and C than in Group A (all *p* < 0.001), whereas no statistically significant differences were found between Group B and Group C for either outcome.

#### 3.1.4. Safety Profile

All the patients were systematically assessed to adverse events (AEs) at every scheduled follow‐up (1, 3, and 6 months). Local infection, nerve injury, skin atrophy, complications of steroids, transient numbness, worsening of pain, and intolerance to ESWT were specific monitoring.

No serious adverse events (SAEs) were reported throughout the study period. Minor, transient AEs included (1) transient medial heel numbness of less than 24 h in two patients (one in Group B and one in Group C), which resolved without sequelae; (2) mild postprocedural pain worsening in 48 h following ESWT in five patients (two in Group A, two in Group B, and one in Group C), which resolved with conservative treatment (rest and ice packs).

There were no infection, skin atrophy, residual neurological deficit, and systemic effects of steroids. None of the patients dropped out because of intolerance. These findings suggest that both injection modalities in conjunction with ESWT have good safety profile in the treatment of PF.

In order to further determine the effectiveness of treatment, we evaluated the 6‐month Modified MacNab Criteria. As shown in Table 3, an excellent–good rate was significantly higher in Group C and Group B compared to that in Group A (*p* < 0.001). Moreover, patient subjective satisfaction was collected at the 3‐ and 6‐month follow‐ups. The combined therapy groups (B and C) reported satisfaction rates above 85%, significantly outperforming the monotherapy group (Group A). These findings corroborate the VAS and AOFAS results, confirming the robust clinical utility of the combination regimens.

**TABLE 3:** Clinical efficacy and adverse events at 3‐ and 6‐months post‐treatment.

Group	*n*	Response rate	6‐month modified MacNab criteria, *n* (%)				6‐month excellent–good rate, *n* (%)
		(≥ 50% reduction in VAS), *n* (%)					
			Excellent	Good	Fair	Poor	
Group A	76	19 (25.00%)	5 (6.58%)	34 (50.75%)	20 (26.32%)	7 (9.21%)	39 (51.32%)
Group B	66	64 (96.97%)	25 (37.88%)	32 (48.48%)	7 (10.61%)	2 (3.03%)	57 (86.36%)
Group C	67	67 (100.00%)	43 (64.18%)	20 (29.85%)	4 (5.97%)	0 (0.00%)	63 (94.03%)
*χ* ^2^		129.094					27.841
*p*		**< 0.001** ^∗^					**< 0.001** ^∗^

*Note:* Bold values represent *p* < 0.05, indicating statistically significant differences.

^∗^Significant as *p* value < 0.05.

### 3.2. Multivariable Linear Regression Analysis of ΔVAS Determinants

A multiple linear regression model was created using the enter method. ΔVAS (baseline VAS‐3 month follow‐up VAS) was the dependent variable, and the independent variables were age, sex, symptom duration, root bone spur length (corrected from the original typo calcaneal spur), diabetes (no/yes), smoking (no/yes), daily standing time, baseline VAS, baseline AOFAS, and BMI. The overall model demonstrated statistical significance (*F* = 34.766, *p* < 0.001), with an adjusted *R* ^2^ of 0.678, indicating that the model explained 67.8% of the variance in ΔVAS. Regression coefficient analysis revealed significant negative associations between symptom duration and ΔVAS, as well as between calcaneal spur length and ΔVAS. Baseline AOFAS was positively associated with ΔVAS, whereas baseline VAS was negatively associated with it. Age, sex, BMI, diabetes status, smoking history, and daily standing time did not reach statistical significance in this model (all *p* < 0.05) (Table 4).

**TABLE 4:** Multivariable linear regression of △VAS.

	*B*	SE	*β*	*t*	*p*	95% CI	VIF
Age (years)	0.007	0.005	0.066	1.365	0.174	−0.003∼0.017	1.494
Gender (male vs female)	0.102	0.105	0.044	0.977	0.330	−0.104∼0.309	1.332
Duration (months)	−0.013	0.006	−0.11	−2.253	**0.025** ^∗^	−0.025∼‐0.002	1.534
Spur length	−0.061	0.015	−0.166	−4.056	**< 0.001** ^∗^	−0.09∼‐0.031	1.086
Diabetic status	−0.072	0.179	−0.02	−0.400	0.690	−0.424∼0.281	1.638
Smoking status	0.058	0.127	0.021	0.455	0.650	−0.193∼0.308	1.324
Upright time							
< 4 h/day	0.178	0.131	0.066	1.359	0.176	−0.08∼0.435	1.513
4–8 h/day	0.095	0.109	0.041	0.874	0.383	−0.12∼0.31	1.443
> 8 h/day	Ref	Ref	Ref	Ref	Ref	Ref	Ref
Baseline VAS Score	−0.249	0.082	−0.164	−3.033	**0.003** ^∗^	−0.411∼‐0.087	1.885
Baseline AOFAS Score	0.075	0.006	0.812	13.496	**< 0.001** ^∗^	0.064∼0.086	2.340
BMI							
Low risk for obesity (below 18.5)	−0.309	0.312	−0.041	−0.990	0.323	−0.925∼0.306	1.106
Risk for nutritional deficiency (18.5–22.9)	Ref	Ref	Ref	Ref	Ref	Ref	Ref
Moderate risk for obesity (23–27.4)	−0.07	0.105	−0.03	−0.670	0.504	−0.277∼0.137	1.309
High risk for obesity (27.5 and above)	0.189	0.176	0.049	1.076	0.283	−0.158∼0.535	1.356

*Note:* Bold values represent *p* < 0.05, indicating statistically significant differences.

^∗^Significant as *p* value < 0.05.

### 3.3. Subgroup Analysis: Impact of Heel Spur Length on Efficacy in Chronic Patients

In order to explore the effect of spur length on the early efficacy of treatment regimens in chronic patients, we divided the subgroup of patients with disease duration longer than 3 months into three subgroups according to the spur length measured on lateral foot x‐ray films. The difference of VAS score before and 3 months after treatment (ΔVAS) was used as the efficacy index. Two‐way analysis of variance was used to test the main effect of treatment regimen and spur length and their interaction. If the interaction was significant, simple effect analysis and Bonferroni correction were used for post hoc pairwise comparisons.

Among the 108 chronic patients, the baseline data of the short, medium, and long spur subgroups were balanced (all *p* < 0.05). Two‐way analysis of variance showed that there was a significant interaction between treatment regimen and spur length on ΔVAS (*F* (4, 98) = 11.137, *p* < 0.001).

Simple effects analysis showed that:

The difference in the ΔVAS of group A was less than that of Group B and Group C (*p* < 0.001) as well as the difference between the VAS of Group B and Group C (*p* < 0.001).

In the middle spur subgroup, VAS of Group A was less than that of Group B and Group C (both *p* < 0.001), but there was no significant difference between Group B and Group C.

Group A of the long spur subgroup had a lower value of VAS than Group B (*p* < 0.001) and Group C (*p* < 0.006), whereas the value of VAS of Group B was greater than that of Group C (*p* < 0.002) (Table 5).

**TABLE 5:** Subgroup analysis of VAS score improvement (ΔVAS) at 3 months in chronic patients, stratified by heel spur length.

	Subgroup (chronic patients)	Group A (*n* = 36)	Group B (*n* = 37)	Group C (*n* = 35)
ΔVAS	All Patients (*n* = 108)			
	Heel Spur < 2.5 mm (*n* = 46)	2.55 ± 0.47	3.20 ± 0.31^#^	4.10 ± 0.44^#$^
	2.5 mm ≤ Heel Spur ≤ 5 mm (*n* = 32)	2.53 ± 0.44	3.18 ± 0.27^#^	3.29 ± 0.29^#^
	Heel Spur > 5 mm (*n* = 30)	1.81 ± 0.65	3.09 ± 0.55^#^	2.38 ± 0.25^#$^
		*F* (4, 98) = 11.137, **p** < 0.001		

*Note:* ΔVAS was calculated as (Baseline VAS ‐3‐month VAS). Data are presented as Mean ± SD. Bold values represent *p* < 0.05, indicating statistically significant differences.

^#^Differences exist between the group and Group A.

^$^Differences exist between the group and Group B.

### 3.4. One‐Month Clinical Efficacy: Modified MacNab Response and Satisfaction Rates

At the 1‐month post‐treatment follow‐up, clinical efficacy was evaluated using the Modified MacNab Criteria.

The MacNab‐defined response rate (defined as achieving at least a “Fair” outcome) was 25.00% in Group A, 96.97% in Group B, and 100.00% in Group C. Pearson chi‐squared test revealed a statistically significant overall intergroup difference (*χ* ^2^ = 129.094, *p* < 0.001). Post hoc pairwise comparisons with the Bonferroni correction (adjusted significance threshold: *p* < 0.0167 for three pairwise comparisons) showed that the response rate in Group A was significantly lower than those in Group B and Group C (both adjusted *p* < 0.001), while no significant difference was detected between Group B and Group C (adjusted *p* < 0.0167).

The Modified MacNab Criteria were used to determine the satisfaction as a good or excellent result. Group A had an overall rate of 59.09%, Group B had an overall rate of 86.36%, and Group C had an overall rate of 94.03% with significant differences between the groups (*χ* ^2^ = 27.841, *p* < 0.001). It was also found that in the pairwise comparisons, the level of satisfaction of the members of Group A was significantly lower than those of both Group B and Group C (both adjusted *p* < 0.001), but not between Group B and Group C (Table 3).

## 4. Discussion

Plantar fasciopathy represents a heterogeneous heel‐pain condition blending mechanical entheseal degeneration and nociplastic pain components, which partly explains variable responses to ESWT across patient subgroups [19, 20]. While ESWT promotes tissue remodeling at the plantar fascia insertion, standalone ESWT frequently fails to achieve satisfactory symptom relief in chronic cases complicated by peripheral and central pain sensitization [21–23]. Ultrasound‐guided plantar FI and tarsal‐canal TNB target distinct pain pathways, yet head‐to‐head comparative evidence for combining these interventions with ESWT remains limited [24–26]. More importantly, few existing studies have explored whether simple radiographic markers can stratify optimal interventional strategies for individual patients. Acute and chronic plantar fasciopathy differ fundamentally in the underlying pain biology, which shapes therapeutic trajectories. Acute presentations are dominated by reversible entheseal microtrauma and local soft‐tissue edema, where endogenous tissue repair capacity remains largely preserved. Even without neural or local injectable adjuvants, ESWT alone can facilitate healing, consistent with comparable long‐term functional restoration across treatment arms in acute patients [24, 27]. The early symptomatic benefit observed under combined injection‐ESWT regimens reflects accelerated symptom suppression rather than durable superiority of multimodal approaches for acute disease.

Notably, in acute PF, intergroup differences in VAS persisted at 3 months, whereas AOFAS scores had already converged across groups, suggesting that functional restoration precedes complete pain resolution in acute disease. This dissociation may reflect that once soft‐tissue edema subsides, patients regain functional capacity despite residual low‐level pain, which gradually resolves with continued endogenous tissue repair.

Chronic plantar heel pain, by contrast, frequently transitions toward mixed nociceptive–nociplastic pathophysiology, with high rates of peripheral sensitization and central pain amplification reported in contemporary cohorts [19]. TNB exerts rapid analgesia primarily by interrupting afferent nociceptive signaling from the tarsal canal, breaking self‐sustained neurogenic pain loops, which accounts for its prominent short‐term clinical advantage [28]. Plantar fascia injection, conversely, targets the degenerative enthesis itself, mitigating local mechanical‐driven nociception; its therapeutic effects build gradually as connective‐tissue repair proceeds, diminishing early intergroup differences at later follow‐ups. This mechanistic divergence may offer a plausible rationale for the more pronounced early improvement observed with TNB, whereas both combination approaches converge toward comparable pain and functional outcomes by the 6‐month follow‐up.

From a clinical significance perspective, the significantly higher MCID achievement rates for both VAS and AOFAS in the combined‐therapy groups among chronic patients confirm that the observed statistical differences translate into clinically meaningful improvements rather than marginal changes of questionable patient relevance. This is further corroborated by the markedly higher Modified MacNab excellent–good rates and responder rates (≥ 50% VAS reduction) in Groups B and C, collectively supporting the clinical utility of adding injectable adjuvants to ESWT for chronic PF.

The clinical relevance of calcaneal spurs in plantar fasciopathy remains debated in recent literature: spurs can be incidentally detected in asymptomatic feet, yet accumulating data indicate that spur dimension correlates with pain phenotypes and treatment responsiveness [29, 30]. Our subgroup findings support a conceptual model where spur size reflects the relative contribution of two pain drivers: neurogenic sensitization versus mechanical entheseal traction. Small spurs (< 2.5 mm) imply pain predominantly rooted in tibial‐nerve‐mediated nociplastic mechanisms, rendering TNB‐ESWT more favorable. Intermediate spur sizes correspond to overlapping contributions of both pathways, producing equivalent outcomes for the two injection‐combined modalities. Larger spurs (> 5 mm) signify predominant mechanical stress at the fascia‐calcaneus insertion, making local fascia‐targeted injection more appropriate. Multivariate regression further identified spur length as an independent predictor of pain relief magnitude, reinforcing its value as a practical stratification marker. This constitutes the key novelty of our work: moving beyond purely empirical comparison of injection techniques toward a radiographic‐guided mechanistic treatment selection paradigm, which has seldom been emphasized in prior comparative interventional studies.

Beyond spur length, the multivariable model yielded additional prognostic insights. Symptom duration emerged as a negative predictor of pain relief, consistent with the notion that prolonged illness promotes peripheral and central sensitization, thereby diminishing treatment responsiveness—an observation that supports early intervention before chronicity becomes established. Baseline AOFAS was positively associated with ΔVAS, suggesting that preserved pretreatment functional capacity may indicate greater rehabilitative reserve and hence better treatment response. Conversely, higher baseline VAS predicted smaller pain reduction, possibly reflecting a ceiling effect in achievable improvement or more severe underlying nociplastic sensitization. The model’s adjusted *R* ^2^ of 0.678 indicates that the included variables account for a substantial proportion of variance in treatment response, reinforcing the robustness of the predictor framework.

Regarding safety, the two cases of transient medial heel numbness—one each in the FI and TNB groups—likely represent brief irritation of small sensory nerve branches by the injectate or needle trajectory rather than true nerve injury, given their complete resolution within 24 h. The five cases of post‐ESWT pain exacerbation are consistent with the well‐documented transient inflammatory response following shock wave treatment. The absence of SAEs across all groups supports the feasibility and safety of both injectable–ESWT combination protocols in routine clinical practice. Collectively, our findings offer a practical treatment algorithm: for acute PF, ESWT monotherapy may suffice given comparable long‐term outcomes, whereas for chronic PF, combination therapy guided by heel spur length—TNB for small spurs and FI for large spurs—may optimize individualized outcomes.

Several limitations of the present study should be acknowledged. First, this was a single‐center retrospective cohort study. Randomized group allocation was not performed; therefore, selection bias could not be completely eliminated and some confounding variables could not be fully balanced across groups. Second, primary outcome measurements depended on the VAS and AOFAS scales. Patient‐reported foot‐specific instruments including FFI and FAAM questionnaires were not incorporated into the assessment protocol, which creates certain limitations in outcome evaluation. In addition, the follow‐up duration was limited to 6 months; long‐term prognostic data are absent, and recurrence rates across the three treatment arms could not be evaluated. Given these above‐mentioned drawbacks, further multicenter prospective randomized controlled trials are warranted to validate the conclusions from our present investigation.

## 5. Conclusions

This research proved that the ESWT and ultrasound‐guided interventional therapy can be used to greatly enhance the clinical effectiveness of chronic PF. TNB is useful in speeding up early functional recovery. More importantly, the length of heel spur is a useful predictor of the choice of combination treatment: for chronic PF, patients with small spur are more suitable for TNB, while patients with large spur may benefit more from fascial injection.

## Funding

The authors received no specific funding for this work.

## Conflicts of Interest

The authors declare no conflicts of interest.

## Data Availability

The data that support the findings of this study are available from the corresponding author upon reasonable request.

## References

[1] Zhao P. , He Y. , Li M. , Wang J. , Wang R. , and Cui X. , Comparative Efficacy and Acceptability of Different Intensity Levels of Extracorporeal Shock Wave Therapy in Adults With Plantar Heel Pain: A Systematic Review and Network Meta-Analysis, Pharmacy Management R. (December 2025) 17, no. 12, 1481–1493, 10.1002/pmrj.13417. 40709373

[2] Yu J. , An J. , Wang S. et al., Endoscopic Plantar Fascia Release via Dual Medial Deep Fascia Approach for Refractory Plantar Fasciitis: An Effective, Safe, and Rapid Surgical Approach, International Orthopaedics. (June 2025) 49, no. 6, 1373–1385, 10.1007/s00264-025-06499-z. PMC12075328 40095072

[3] Tung W. S. , Daher M. , Covarrubias O. , Herber A. , and Gianakos A. L. , Extracorporeal Shock Wave Therapy Shows Comparative Results With Other Modalities for the Management of Plantar Fasciitis: A Systematic Review and Meta-Analysis, Foot and Ankle Surgery. (June 2025) 31, no. 4, 283–290, 10.1016/j.fas.2024.11.005. 39572278

[4] Gollwitzer H. , Saxena A. , DiDomenico L. A. et al., Clinically Relevant Effectiveness of Focused Extracorporeal Shock Wave Therapy in the Treatment of Chronic Plantar Fasciitis: A Randomized, Controlled Multicenter Study, Journal of Bone and Joint Surgery. (May 2015) 97, no. 9, 701–708, 10.2106/JBJS.M.01331. 25948515

[5] Ong S. S. L. , Mao D. W. , Socklingam R. K. , Moo I. H. , and Kon K. K. C. , A Prospective Randomized Controlled Trial Comparing Extracorporeal Shockwave Therapy and Physiotherapy in the Treatment of Acute Plantar Fasciitis, Journal of Orthopaedics. (March 2025) 70, 25–28, 10.1016/j.jor.2025.03.015. PMC11982301 40225063

[6] Kuzu Ö. , Çakır M. , Aras B. , Ata A. M. , and Kesikburun B. , Prospective Comparative Study Between Extracorporeal Shock Wave Therapy and Ultrasound-Guided Ozone (O2-O3) Injection in Patients With Plantar Fasciitis, Journal of Foot and Ankle Surgery. (July 2025) 4, no. 25, S1067–S2516, 10.1053/j.jfas.2025.05.015. 40618836

[7] McMillan A. M. , Landorf K. B. , Gilheany M. F. , Bird A. R. , Morrow A. D. , and Menz H. B. , Ultrasound Guided Corticosteroid Injection for Plantar Fasciitis: Randomised Controlled Trial, BMJ. (May 2012) 344, no. may22 1, 10.1136/bmj.e3260. 22619193

[8] Peña-Martínez V. M. , Acosta-Olivo C. , Simental-Mendía L. E. et al., Effect of Corticosteroids Over Plantar Fascia Thickness in Plantar Fasciitis: A Systematic Review and Meta-Analysis, Physician and Sportsmedicine. (June 2024) 52, no. 3, 217–228, 10.1080/00913847.2023.2223673. 37293970

[9] David J. A. , Sankarapandian V. , Christopher P. R. , Chatterjee A. , and Macaden A. S. , Injected Corticosteroids for Treating Plantar Heel Pain in Adults, Cochrane Database of Systematic Reviews. (June 2017) 6, no. 6, 10.1002/14651858.CD009348.pub2. PMC6481652 28602048

[10] Llurda-Almuzara L. , Labata-Lezaun N. , Meca-Rivera T. et al., Is Dry Needling Effective for the Management of Plantar Heel Pain or Plantar Fasciitis? An Updated Systematic Review and Meta-Analysis, Pain Medicine. (July 2021) 22, no. 7, 1630–1641, 10.1093/pm/pnab114. 33760098

[11] Analay P. , Abdulsalam A. J. , Kara M. , and Özçakar L. , Ultrasound-Guided Injections for Plantar Fasciitis, Turkish Journal of Physical Medicine and Rehabilitation. (October 2024) 71, no. 2, 260–261, 10.5606/tftrd.2024.15430. PMC12305630 40740175

[12] Tütüncü M. N. , Sekizkardeş M. , Şahin E. , and Karaismailoğlu B. , Comparative Efficacy of Corticosteroid Injection, Extracorporeal Shock Wave Therapy, and Radiofrequency Ablation for Chronic Plantar Fasciitis: A Prospective Randomized Controlled Trial, BMC Musculoskeletal Disorders. (January 2026) 27, no. 1, 10.1186/s12891-026-09542-1. PMC12918559 41593558

[13] Aktan Ç. and Aktan C. , Comparative Effectiveness of Ultrasound-Guided Corticosteroid Injection, Radiofrequency Ablation, and Their Combination for Recalcitrant Plantar Fasciitis: A Retrospective Cohort Study, Journal of Foot and Ankle Research. (September 2025) 18, no. 3, 10.1002/jfa2.70080. PMC12411256 40908556

[14] Digiovanni B. F. , Nawoczenski D. A. , Malay D. P. et al., Plantar Fascia-Specific Stretching Exercise Improves Outcomes in Patients With Chronic Plantar Fasciitis: a Prospective Clinical Trial With Two-Year Follow-Up, Journal of Bone and Joint Surgery. (2006) 88, no. 8, 1775–1781, 10.2106/00004623-200608000-00013. 16882901

[15] Shalaby M. , Sanoja A. , and Rosselli M. , Posterior Tibial Nerve Block for the Treatment of Plantar Fasciitis in the Emergency Department, Journal of Emergency Medicine. (November 2023) 65, no. 5, e441–e443, 10.1016/j.jemermed.2023.05.025. 37739850

[16] Çağlar Okur S. and Aydın A. , Comparison of Extracorporeal Shock Wave Therapy With Custom Foot Orthotics in Plantar Fasciitis Treatment: A Prospective Randomized One-Year Follow-Up Study, Journal of Musculoskeletal and Neuronal Interactions. (June 2019) 19, no. 2, 178–186. PMC6587088 31186388

[17] Aykaç B. , Dinçz M. , Bayrak H. Ç. , and Karasu R. , Quantitative Radiographic Morphology of Posterior Calcaneal Spurs Independently Predicts Patient-Centered Outcomes After Extracorporeal Shockwave Therapy for Insertional Achilles Tendinopathy: An MCID and PASS Analysis, Journal of Clinical Medicine. (February 2026) 15, no. 4. 10.3390/jcm15041538 PMC12942205 41753225

[18] Gong X. F. , Sun N. , Li H. et al., Modified Chevron Osteotomy With Distal Soft Tissue Release for Treating Moderate to Severe Hallux Valgus Deformity: A Minimal Clinical Important Difference Values Study, Orthopaedic Surgery. (July 2022) 14, no. 7, 1369–1377. 10.1111/os.13242 PMC9251292 35633110

[19] Demir K. G. , Melek Aykut Selçuk M. , and Öztürk E. A. , Frequency of Central Sensitization and Nociplastic Pain in Patients With Plantar Fasciitis: Central Sensitization and Nociplastic Pain in Plantar Fasciitis, International Orthopaedics. (May 2025) 49, no. 5, 1091–1099, 10.1007/s00264-025-06462-y. PMC12003458 40014141

[20] Chen Y. , Lyu K. , Lu J. et al., Biological Response of Extracorporeal Shock Wave Therapy to Tendinopathy In Vivo (Review), Frontiers in Veterinary Science. (July 2022) 9, 10.3389/fvets.2022.851894. PMC9356378 35942112

[21] Wheeler P. C. , Up to a Quarter of Patients With Certain Chronic Recalcitrant Tendinopathies may Have Central Sensitisation: A Prospective Cohort of More Than 300 Patients, British Journal of Pain. (August 2019) 13, no. 3, 137–144, 10.1177/2049463718800352. PMC6613072 31308939

[22] Chang C. W. , Wang Y. C. , Hou W. H. , Lee X. X. , and Chang K. F. , Medial Calcaneal Neuropathy is Associated With Plantar Fasciitis, Clinical Neurophysiology. (January 2007) 118, no. 1, 119–123, 10.1016/j.clinph.2006.09.028. 17095287

[23] Takla M. K. N. and Rezk S. S. R. , Clinical Effectiveness of Multi-Wavelength Photobiomodulation Therapy as an Adjunct to Extracorporeal Shock Wave Therapy in the Management of Plantar Fasciitis: A Randomized Controlled Trial, Lasers in Medical Science. (April 2019) 34, no. 3, 583–593, 10.1007/s10103-018-2632-4. 30194553

[24] Li S. , Wang K. , Sun H. et al., Clinical Effects of Extracorporeal Shock-Wave Therapy and Ultrasound-Guided Local Corticosteroid Injections for Plantar Fasciitis in Adults: A Meta-Analysis of Randomized Controlled Trials, Medicine (Baltimore). (December 2018) 97, no. 50, 10.1097/MD.0000000000013687. PMC6320028 30558080

[25] Sobey J. H. and Franklin A. , Ultrasound-Guided Tibial Nerve Block for Definitive Treatment of Tarsal Tunnel Syndrome in a Pediatric Patient, Regional Anesthesia and Pain Medicine. (June 2016) 41, no. 3, 415–416, 10.1097/AAP.0000000000000384. 27093282

[26] Kane D. , Greaney T. , Bresnihan B. , Gibney R. , and FitzGerald O. , Ultrasound Guided Injection of Recalcitrant Plantar Fasciitis, Annals of the Rheumatic Diseases. (June 1998) 57, no. 6, 383–384, 10.1136/ard.57.6.383. PMC1752613 9771217

[27] Zhang L. , Fu X. B. , Chen S. , Zhao Z. B. , Schmitz C. , and Weng C. S. , Efficacy and Safety of Extracorporeal Shock Wave Therapy for Acute and Chronic Soft Tissue Wounds: A Systematic Review and Meta-Analysis, International Wound Journal. (August 2018) 15, no. 4, 590–599, 10.1111/iwj.12902. PMC7949564 29675986

[28] Ritt M. W. J. , Koning H. , van Dalen B. V. , and Ter Meulen B. C. , Tibial Nerve Block as Treatment of Chronic Foot Pain, Anesthesiology and Pain Medicine. (January 2023) 13, no. 1, 10.5812/aapm-131180. PMC10363362 37489169

[29] Okçu M. , Tuncay F. , Koçak F. A. , Erden Y. , Ayhan M. Y. , and Kaya S. S. , Do the Presence, Size, and Shape of Plantar Calcaneal Spurs Have Any Significance in Terms of Pain and Treatment Outcomes in Patients With Plantar Fasciitis?, Turkish Journal of Medical Sciences. (February 2023) 53, no. 1, 413–419, 10.55730/1300-0144.5598. PMC10387834 36945957

[30] Kuyucu E. , Koçyiğit F. , and Erdil M. , The Association of Calcaneal Spur Length and Clinical and Functional Parameters in Plantar Fasciitis, International Journal of Surgery. (September 2015) 21, 28–31, 10.1016/j.ijsu.2015.06.078. 26184993

